# Commentary: Rapid and facile preparation of giant vesicles by the droplet transfer method for artificial cell construction

**DOI:** 10.3389/fbioe.2022.1037809

**Published:** 2022-10-14

**Authors:** Pasquale Stano

**Affiliations:** Department of Biological and Environmental Sciences and Technologies (DiSTeBA), University of Salento, Lecce, Italy

**Keywords:** synthetic cells, artificial cells, protocells, lipid vesicles, liposomes, liposome technology, droplet transfer method, inverted emulsion method

## 1 The “droplet transfer” (or “inverted emulsion” or “emulsion transfer” or “phase transfer”) method

When considered within the well-established field of liposome technology, the droplet transfer method (DTm) might appear as a rather niche technique, but it has literally revolutionized experimental approaches to bottom-up Artificial Cells (ACs) (Stano, 2019). The DTm was firstly used by Vincent Noireaux and Albert Libchaber in their renowned 2004 “bioreactor” paper (Noireaux and Libchaber, 2004) and since then it has been widely applied to the construction of several types of ACs. This method is at the basis of several successful studies, which are not commented here for space limitations. A general discussion on the method can be found in Walde et al. (2010) and in Chapter 1 of “*The Giant Vesicle Book*” (Dimova et al., 2020).

The timely investigation presented in *Frontiers on Bioengineering and Biotechnology* by Shimane and Kuruma (2022), to which this commentary is dedicated, proves the importance and the utility of the DTm in order to prepare ACs based on giant liposomes (giant lipid vesicles, GVs). In particular, the Authors have illustrated in a quite detailed and practical way the typical operations required to encapsulate complex macromolecular mixtures–such as the PURE system (Shimizu et al., 2001; Shimizu et al., 2005)—inside GVs in physiological and bioactive conditions, and in high yields. The resulting ACs are then competent for *gene expression* (a key AC functionality) thanks to the presence of about 100 different macromolecules and dozens of small MW compounds co-captured in the GV lumen. Proteins will be produced in cell-like fashion, i.e., from within.

This commentary is an opportunity to briefly add some considerations on the DTm. We will highlight its major historical developments, the pros and cons, and the still missing mechanistic details.

## 2 Major historical developments

Demetrios Papahadjopoulos (1934–1998), one of the fathers of liposome research, reported that attempts to prepare liposomes from water-in-oil emulsion droplets date back to the 1970s (Träuble and Grell, 1971; Szoka and Papahadjopoulos, 1980). The aim was the production of conventional small or large unilamellar vesicles (SUVs or LUVs) with an *asymmetric membrane*. This is in principle possible when the two lipid leaflets of a vesicle membrane are assembled sequentially. Membrane asymmetry was also the goal of more recent studies, due to the Weitz group (Pautot et al., 2003a; Pautot et al., 2003b) and to less cited, but prior, investigators (Zhang et al., 1997; Xiao et al., 1998a; Xiao et al., 1998b).

The shift of interest from LUVs to GVs, and from membrane asymmetry to high encapsulation yield coincided with the above-mentioned Noireaux and Libchaber (2004) paper. This double-leap actually made the difference, and brought the DTm to the attention of the AC community. Indeed the DTm, when the *isotonic density gradient* strategy is employed^1^, allows the facile preparation of solute-filled GVs, solving “once for all” (and simultaneously) long standing issues such as efficient macromolecule (co)encapsulation, employment of physiological conditions (concentrated/salty buffers), reduction of the volume of precious solution to be entrapped, reduction of the preparation time, and avoidance of difficult to handle equipment. Moreover, in most cases the resulting GVs appear to be gracefully unilamellar (GUVs) (Chiba et al., 2014).

Subsequent investigations have refined, optimized (Fujii et al., 2014; Moga et al., 2019; Uyeda et al., 2022), scaled-up (Rampioni et al., 2018) and extended the DTm, including the structural analysis of the resulting GVs by flow cytometry (Nishimura et al., 2009), its implementation in microfluidic (Matosevic and Paegel, 2011) or in rotatory capillary devices (Abkarian et al., 2011), but essentially there have been no major noteworthy findings.

## 3 Pros and cons

**FIGURE 1:**
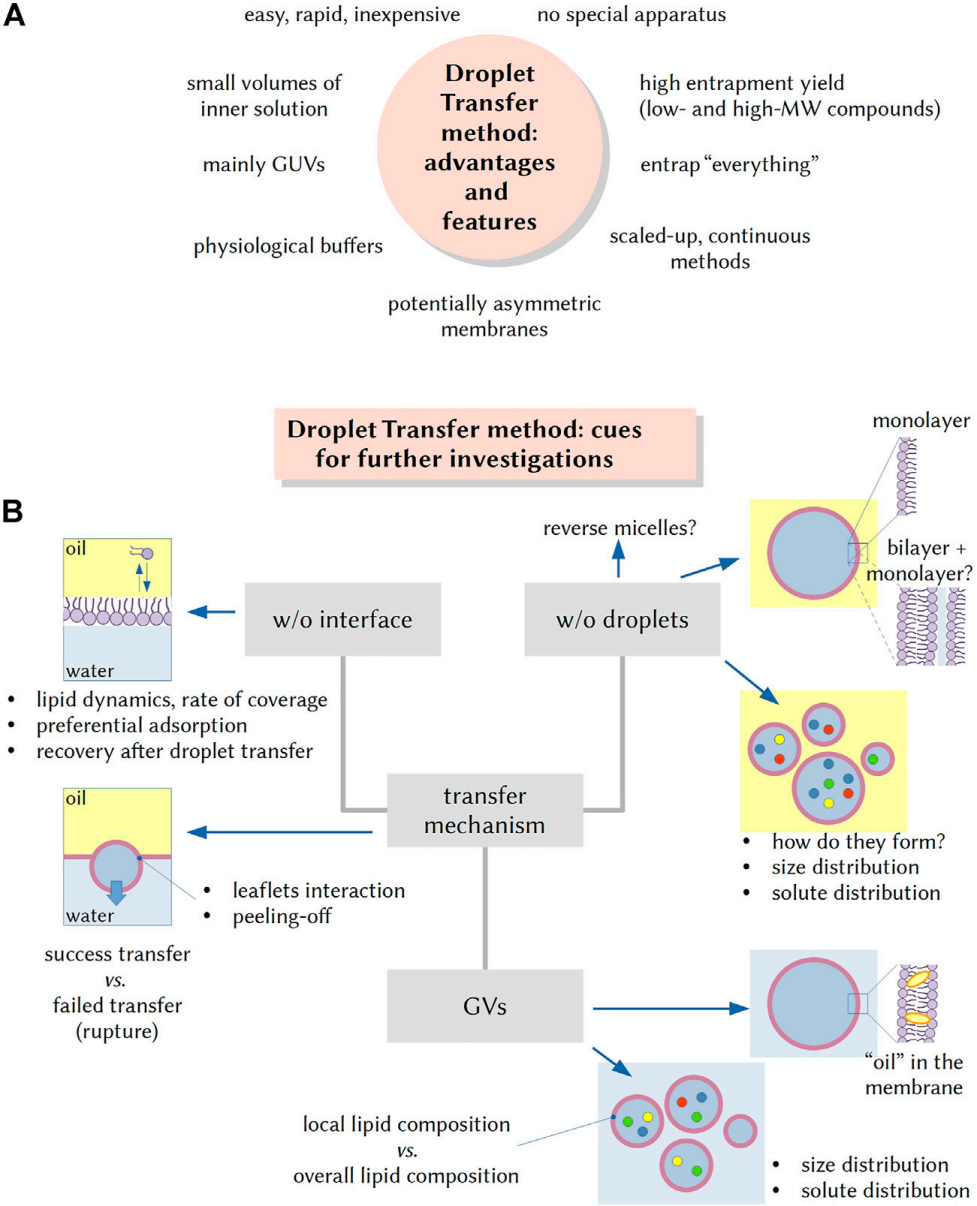
The droplet transfer method (DTm): advantageous features and cues for further investigations. **(A)** The advent of the DTm in AC research is quite attractive as it allows the formation of solute-filled GVs in a quite easy and inexpensive manner. Additional advantages are the rapidity (few minutes), no need of specific equipment, employment of small volumes (especially important when complex mixtures need to be encapsulated in the vesicle lumen), the possibility of using physiological buffers, and the possibility of entrapping very large particles. **(B)** Several experimental steps for carrying out the DTm have been recently investigated and optimized, but open questions remain, and they can be the focus of future investigations. For example, it would be important to know more about the lipid dynamics on the oil/water interface, the rate of interface coverage by lipids and the recovery of lipids in the “naked” area after a w/o droplet has been transfer. What factors affect the fraction of w/o droplets that become GVs? Similarly, the very mechanism of w/o droplet formation (*via* stochastic fragmentation and coagulation) is not well known, as well as the factors determining the droplet size and the solute distribution. Other questions can be: are droplets always covered by a lipid monolayer? What is the amount of residual “oil” in the membrane of resulting GVs? In the case of lipid mixtures, preferential localization of some lipid species on all interfaces (vs those remaining in bulk solution) can occur, and thus, do the obtained GVs have a lipid composition that mirrors the overall lipid composition? Is there any correlation between w/o droplets and GVs size and solute distributions?

The advantages of the DTm have been mentioned above (see also Figure 1A). A further merit consists in the possibility of encapsulating very large particles (e.g., nanoparticles, SUVs/LUVs, organellae, bacteria, etc.) inside GVs. This feature has lead to the easy preparation of nested multi-compartmentalized systems that mimic eukaryotic cells. A pregnant example refers to ACs endowed with natural or artificial energy-producing organellae in their lumen (Berhanu et al., 2019; Altamura et al., 2021). Another valuable feature concerns the reconstitution of membrane proteins from within, in order to obtain otherwise unfeasible physiological-like orientations (Yanagisawa et al., 2011; Altamura et al., 2017).

The most-often repeated criticism pertains to the possible residual presence of hydrocarbons in the GVs membrane. Researchers interested in studying phenomena strictly dependent on biophysical/biochemical properties of membranes are understandably worried about this issue (e.g., membrane permeability and mechanical properties, dynamics of proteins embedded into the membrane) (e.g., Campillo et al., 2013; Kamiya et al., 2016; Faizi et al., 2022). The “mineral oils” typically used in the DTm actually are cheap heterogeneous mixture of linear, branched, and possibly cyclic hydrocarbons, which can be adsorbed on or absorbed in the membrane (McIntosh et al., 1980; Richens et al., 2015). Pautot et al. (2003b) already recognized this issue, recalling that it could be alleviated by using specific hydrocarbons (e.g., squalene). Fortunately, there are types of investigations that can be safely carried out disregarding the potential presence of residual oil in the GV membrane (clearly, it depends on the aim of the experiment).

A less frequently mentioned aspect is that DTm is not the best choice in studies intended to explore the *spontaneous* formation of cell-like systems from lipids and solutes. Therefore, when ACs are intended as primitive cell models and the experimental focus is on spontaneous assembly mechanisms in an aqueous environment, other preparation methods should be considered.

## 4 Mechanistic details

The ever increasing number of reports that exploit the production of ACs based on the DTm contrasts with the sporadic study about its mechanistic details. Pautot *et al.* (2003ab) reported several insightful observations, but because their protocol is quite different from the ones commonly used today, more investigations are needed (see Figure 1B). An elegant study made with a 90° tilted microscope (in order to observe the dynamic of droplet transfer) came from the group of Yoshikawa (Ito et al., 2013).

For example, open questions concern the mechanism of formation and the structure of the w/o droplets (always surrounded by a monolayer?) and the distribution of solutes therein. The dynamics of lipids at the interface between the emulsion phase and the aqueous outer phase and the very mechanism of bilayer formation *via* adhesion of the two lipid leaflets, during the transfer, are key aspects too. Moreover, the case when lipid mixtures are used is not well studied (as well as the usage of non-phospholipid amphiphiles): does the composition of the resulting GV membranes mirrors the overall bulk composition of lipids used in the preparation? (Some lipids could be preferentially included in the membrane).

## Footnotes

1 The isotonic density gradient was introduced, to the best of our knowledge, by Takagi and collaborators (Hamada et al., 2008; but see also Hadorn and Eggenberger Hotz, 2009), providing a key modification that has definitely led a reliable GV production method. It consists in the usage of isotonic sucrose and glucose solutions (e.g., 200 mM or higher), respectively, as inner and outer aqueous solutions, in order to facilitate the droplet transfer from the top oil phase to the bottom aqueous phase. High concentrations of sugars, however, can affect membrane properties (Andersen et al., 2011). Träuble and Grell (1971) employed instead CsCl gradients, but the resulting vesicles were osmotically sensitive.
